# Clinical efficacy of PD-1 inhibitor combined with radiotherapy in a multi-drug resistant patient with liver metastasis from gastric cancer

**DOI:** 10.3389/fsurg.2023.1101294

**Published:** 2023-04-20

**Authors:** Judi Xu, Hedai Liu, Guoying Ni, Yan Huang, Ying Huang, Hongxiang Liang, Yufeng Ni, Qian Huang, Zhiyong Yang

**Affiliations:** Department of Oncology, Xinhua Hospital Chongming Branch (Chongming Hospital Affiliated to Shanghai University of Medicine and Health Sciences), Shanghai, China

**Keywords:** advanced gastric cancer, combined therapy, PD-1 inhibitor, radiation and chemotherapy, anti-angiogenesis therapy

## Abstract

A 54-year-old male was diagnosed with extensive liver metastasis and small nodule metastasis in the lungs from gastric adenocarcinoma [Her-2 (−)]. The patient achieved significant partial response (PR) after chemotherapy combined with anti-angiogenesis therapy but developed progressive disease (PD) after 5 months. Then, the chemotherapeutic and anti-angiogenic drugs were replaced. Meanwhile, the delivery route of some chemotherapeutic drugs was changed, and some chemotherapeutic drugs were given *via* transcatheter arterial chemoembolization (TACE) to achieve PR, and PD developed after 3 months of remission maintenance. During chemotherapy combined with anti-angiogenesis, the application of programmed cell death-1 (PD-1) inhibitor achieved PR again and maintained for 5 months before disease progression. The progression of the lesions in the left lobe of the liver and the hepatic hilar lymph nodes was significant. Hence, chemotherapy was terminated and gamma stereotactic body radiation therapy (SBRT) was performed on left lobe lesions and hilar lymph nodes. The lesions both inside and outside the radiation field regressed significantly, reaching PR and abscopal effects. The immune-related adverse events (irAEs) occurred, including erythema and black and luster hair. The abscopal effects of lesion reduction in the radiation field and the enhancement of the immune function stimulated by radiation are a highlight of the combination of radiation and immunotherapy. In the end, the patient died of gastrointestinal failure, with overall survival of 18 months.

## 1. Introduction

Gastric cancer (GC) is one of the most common malignant tumors worldwide and ranks third for cancer incidence among the male population in China, and what's worse, nearly half of the patients with GC are diagnosed at advanced stages (1). Usually, the natural history of liver metastasis from GC is 3 to 6 months. Currently, systemic therapy is recommended for patients with advanced GC. Chemotherapy combined with targeted therapy and/or immunotherapy contributes to prolonging the survival time. In particular, the synergistic effects of immunotherapy and palliative radiotherapy have been reported to produce promising survival benefits (2). However, there are relatively few reports on the treatment of GC patients with liver metastasis *via* the combination of immunotherapy and palliative radiotherapy.

## 2. Case presentation

### 2.1. Clinical data

**Figure 1:**
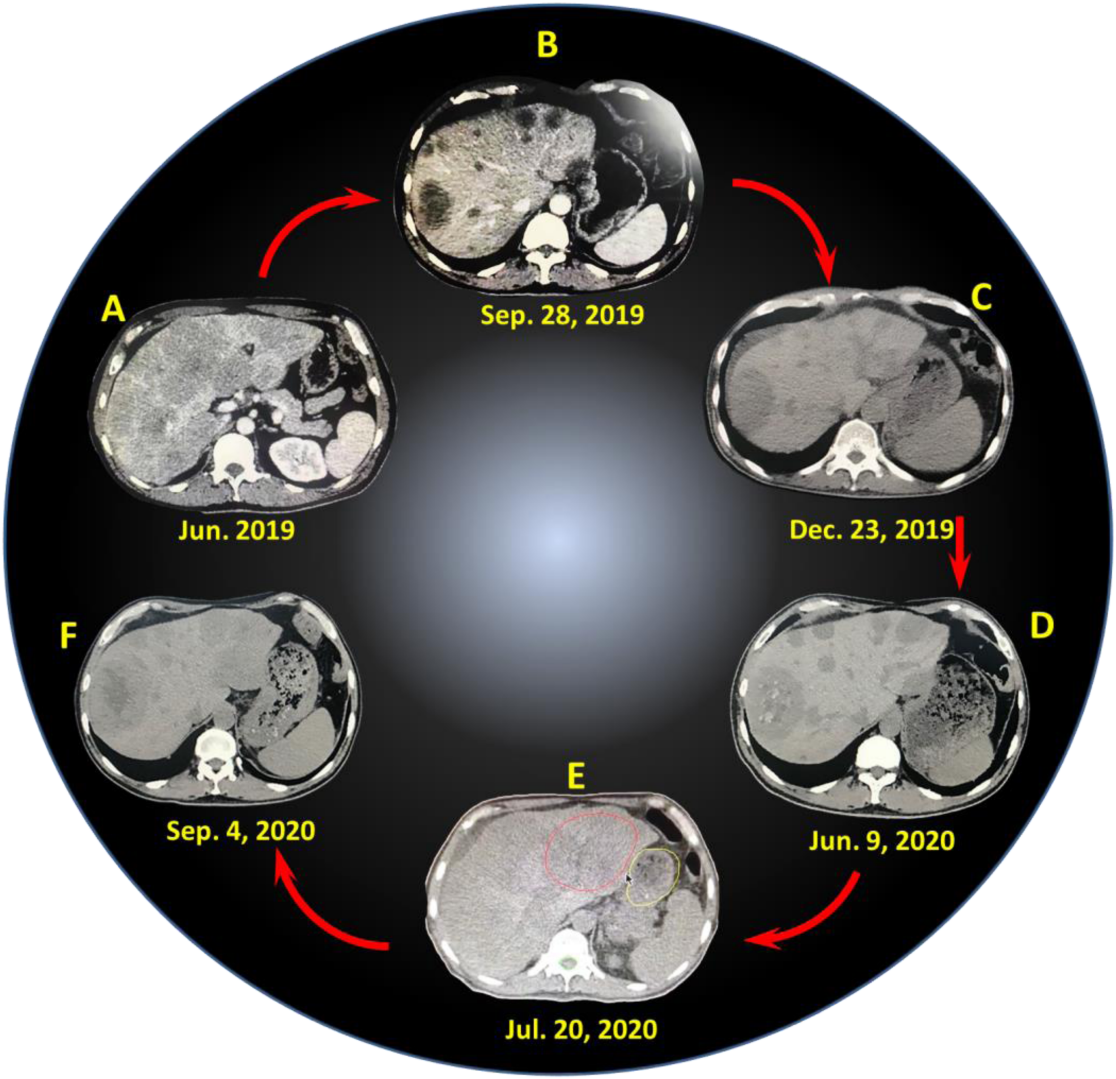
CT images of liver lesions at different time points: (**A**) June 2019 (before treatment), (**B**) September 28, 2019, (**C**) December 23, 2019, (**D**) June 9, 2020, (**E**) July 20, 2020, (**F**) September 4, 2020.

A 54-year-old male inadvertently found a mass in the right upper abdomen on June 15, 2019. He had a normal appetite, bowel movements, and urination, but experienced a weight loss of 15 kg within half a year. Ultrasound image showed multiple space-occupying lesions in the liver, as further confirmed by computed tomography (CT) image (Figure 1A, the magnification image for Supplementary Figure S1), and metastatic liver cancer was considered. The results of endoscopy and biopsy pathology indicated gastric adenocarcinoma [Her-2 (−)]. Physical examination showed the performance status (PS) and NRS score of 0, clear consciousness, mild yellowing of sclera, no superficial enlarged lymph nodes, clear breath sound in both lungs, heart rate of 84 beats per min (BPM), regular rhythm, a flat abdomen, palpable, hard, and fixed liver with clear borders that located in 2 cm–3 cm below ribs and 1 cm–2 cm below the xiphoid process, mild tenderness, liver not palpable under the spleen ribs, negative ascites, and no swelling of both lower extremities. The total bilirubin (TBIL) was 53 μmol/L; the carcinoembryonic antigen (CEA) was 8.01 ng/ml (≤5.09); the alpha-fetoprotein (AFP) was 4.03 IU/ml (≤6.7), and the carbohydrate antigen (CA)199 43 U/ml (0–27). Lung CT showed the existence of multiple nodules, and the size of the largest one was 1.5 cm. Finally, the patient was diagnosed with extensive liver metastasis and small nodule metastasis in the lungs from gastric adenocarcinoma [Her-2 (−)].

### 2.2. Clinical treatment

First of all, the patient received 6 courses of chemotherapy (oxaliplatin + capecitabine + tratinib) in hospital A, and the last treatment was finished on November 25, 2019. During chemotherapy, the patient suffered from grade III myelosuppression involving platelets and grade II myelosuppression involving leukocytes but got improvement after symptomatic treatment. The dose of the last chemotherapy was reduced. The patient was evaluated with partial response (PR) after the first and second courses of chemotherapy (Figure 1B and Supplementary Figure S2), stable disease (SD) after the third and fourth courses, and progressive disease (PD) after the fifth and sixth courses (Figure 1C and Supplementary Figure S3).

On December 3, 2019, the patient was transferred to our hospital (hospital B) and treated with 3 courses of interventional chemotherapy. The first course of treatment was performed *via* transcatheter arterial chemoembolization (TACE) (fluorouracil 1,000 mg + cisplatin 40 mg, pirarubicin 30 mg + lipiodol 10 ml were injected *via* the right hepatic artery). The second and third courses of treatment were performed *via* TACE (fluorouracil 1,000 mg + cisplatin 40 mg, pirarubicin 30 mg + lipiodol 10 ml were injected *via* the right hepatic artery), and no obvious adverse reactions occurred after the intervention. On the second day of the intervention, the patient underwent palliative chemotherapy combined with anti-angiogenesis therapy [docetaxel 140 mg, intravenous drip, day 2; capecitabine 1.0 g, orally, twice a day (day 2–15); anlotinib 12 mg, orally, twice a day (day 2–15)]. Due to disease progression, 6 courses of immunotherapy (carrelizumab 200 mg) was performed starting from February 12, 2020, based on palliative chemotherapy and anti-angiogenesis therapy, and the efficacy was evaluated as PR, SD, or PD per two course during this period (Figure 1D and Supplementary Figure S4). According to the efficacy evaluation, the drugs used were anlotinib, apatinib, docetaxel, fluorouracil, oxaliplatin, and cisplatin, and the last immunotherapy combined with chemotherapy was on July 8, 2020.

After the last chemotherapy, owing to the obvious progression of the liver lesions, especially in the left lobe of the liver and hilar lymph nodes, no significant changes in pulmonary nodules, and no obvious abdominal metastasis, the patient was treated with gamma stereotactic body radiation therapy (SBRT) in hospital C, with the left hepatic lobe and hepatic hilar lesions as the radiation field (Figure 1E and Supplementary Figure S5). On July 20, 2020, SBRT was performed. An OUR-QGD SBRT system was utilized, and a 5-mm slice-distance scan was performed using helical CT. The images were input into the treatment planning system for three-dimensional reconstruction, and the target area was outlined with the left lobe of the liver and the hepatic hilum, and the boundaries did not exceed half of the liver. The prescription dose was based on a 55% isodose curve wrapped around the target area, with a single dose of 3 Gy, once a day, five times a week, for a total of 13 times, and the total dose was 39 Gy. On August 6, 2020, the patient received carrelizumab treatment once. One week after radiotherapy, the patient experienced repeated nausea and vomiting, food intake reduction, fatigue, weight loss, and slight abdominal pain. The reactions of programmed cell death-1 (PD-1) inhibitors were presented, such as fever, rash, erythema with itching, and hair turning black and luster. CT re-examination showed that the irradiation site was the left lobe of the liver (red circle area, in Figure 1E), but the lesions both inside and outside the radiation field were significantly reduced, exhibiting obvious abscopal effects (Figure 1F and Supplementary Figure S6). In addition, the total treatment and relative clinical evaluation of 16 courses was listed and presented in Table S1 (Supplementary Information). However, the patient still experienced frequent nausea and vomiting, which were not alleviated after symptomatic treatment. In addition, the patients did not receive nasal feeding with a jejunal nutrient tube, and his poor physical condition limited the continuation of anticancer therapy. At the end of October 2020, the patient presented with black stool, vomiting of coffee-colored fluid, and ascites. But the patient then became passive, refused examination and active treatment, and was transferred to hospice care. Finally, the patient died of gastrointestinal failure in early November 2020, and the death was considered to be related to gastrointestinal failure and tumor progression.

## 3. Discussion

The continuous advent of new anti-tumor drugs has significantly improved the therapeutic effect of tumors. The tumor growth process is accompanied by the stimulation of tumor angiogenesis, which can deliver nutrients and oxygen to the tumor environment and also remove metabolic byproducts from the tumor environment, thereby further accelerating tumor growth and metastasis. Anti-angiogenesis therapy can inhibit tumor angiogenesis and reduce tumor growth and metastasis. Targeting angiogenesis by inhibition of vascular endothelial-derived growth factors (VEGFs) has been reported to exert considerable therapeutic effects in lung, hepatic, renal, gastric, and colon cancers (3, 4). Li et al. reported that the median overall survival (OS) of patients with metastatic GC (who experienced treatment failure with two or more chemotherapy regimens) treated with apatinib can be prolonged by 2.5 months compared with those treated with a placebo (5). Chemotherapy combined with apatinib can significantly enhance the therapeutic effect of GC and prolong the survival time. The patient in this case achieved significant tumor remission in the first two courses of chemotherapy combined with anti-angiogenesis therapy. Due to disease progression, the anti-angiogenesis drug (tratinib) was changed to anlotinib for combined immunochemotherapy, during which a course of apatinib was used, but it was ineffective.

Immune checkpoint molecules expressed on immune cells can prevent the body from producing effective anti-tumor immune responses, and may also be exploited by tumors to form immune escapes in tumor tissues. Immune checkpoint inhibitors (ICIs) act on immune checkpoints to enhance immune response or relieve immune suppression. The PD-1/PD-L1 pathway is a key immune checkpoint responsible for the negative regulation of the stability and integrity of T-cell immune function. Prior to the CheckMate-649 study (6), many front-line treatments of GC based on PD-1/PD-L1 inhibitors failed to achieve the primary endpoint, making the value of immunotherapy for the front-line treatment of GC questionable. CheckMate-649 had the largest sample size of GC patients to date and compared the therapeutic effect of nivolumab in combination with chemotherapy (O + chemo) or nivolumab in combination with ipilimumab (YO in combination) with that of chemotherapy alone in the untreated patients with HER2-negative, advanced or metastatic GC, gastroesophageal junction cancer, or esophageal adenocarcinoma. A statistically significant difference was observed in the combination of nivolumab and chemotherapy in patients with either PD-L1 combined positive score (CPS) ≥5, ≥1, or all randomized patients. It was also the first global study to prove that the combination of nivolumab and chemotherapy has achieved significant overall survival benefits over chemotherapy. After the progression of the 9th course, the patient obtained a certain degree of therapeutic effect by the immunotherapy with PD-1 inhibitor carrelizumab while receiving chemotherapy and anti-angiogenesis, and maintained a stable state for 7 months.

PD-1/PD-L1 combined with low-dose radiotherapy (LDRT) shows synergistic effects. Kong et al. reported (2) the clinical efficacy and safety of PD-1 inhibitors combined with radiotherapy and granulocyte macrophage-colony stimulating factor (GM-CSF), also known as the PRaG regimen, for refractory patients. Briefly, 16 patients with multi-metastatic solid tumors who experienced first-line chemotherapy failure were recruited and treated with hyper-fractionated radiotherapy (3 doses of 8 Gy or 5 doses of 5 Gy) for each metastatic site, and the median number of metastatic lesions was 7.5. On the second day after radiotherapy, PD-1 inhibitor (200 mg) was intravenously administered once and GM-CSF (200 µg) was subcutaneously injected daily for 2 weeks. All patients completed two or more cycles of triple-combination therapy. After combination therapy, the PD-1 inhibitor was administered for maintenance until disease progression or unacceptable toxicity. The overall response rate (ORR) was 20% and the median progression-free survival (PFS) was 3.3 months (95% CI, 2.3 to 7.2 months) at the time of evaluation, with well-tolerated adverse reactions. Moreover, Fernanda et al. allocated (7) the model mice with local neuroendocrine pancreatic tumors to two groups: the radiation-combined immunotherapy group (RACIM) and the combined immunotherapy group (CIM). They found that 83.5% of mice in the RACIM group showed tumor responses on the 20th day of treatment, with an overall cure rate of 15%, while the mice in the CIM group lacking LDRT did not show a therapeutic effect. These findings suggest that LDRT can effectively control tumor metastasis by recruiting T cells and mobilizing the involvement of innate and adaptive immunity by combining with ICIs. Moreover, LDRT is also demonstrated to reset the immune microenvironment in ovarian cancer patients.

In this case, the patient achieved significant remission of tumor lesions during the period of immunotherapy combined with palliative radiotherapy after the occurrence of drug resistance of targeted chemotherapy and immunotargeted chemotherapy, as manifested by the obvious abscopal effect of lesion reduction outside of the radiation field, activated immune function, as well as immune responses of skin (erythema-like rash) and hair (black and luster). The clinical synergistic effect of radiotherapy and immunotherapy were presented in this case, representing a highlight of the treatment.

## 4. Conclusion

With the development of anti-tumor drugs and the combined application of anti-tumor drugs with different mechanisms, the therapeutic effect of advanced GC has also been significantly improved. The patient in this case is a beneficiary of the combined application of anti-tumor drugs with different mechanisms and also provides a clinical example for enhancing the immune anti-tumor effect of PD-1 inhibitor combined with radiotherapy.

## Data Availability

The raw data supporting the conclusions of this article will be made available by the authors, without undue reservation.
