# Supplementary material for: Clinical efficacy of PD-1 inhibitor combined with radiotherapy in a multi-drug resistant patient with liver metastasis from gastric cancer
Source: Front Surg. 2023 Apr 20;10:1101294. doi: 10.3389/fsurg.2023.1101294 (PMC10157034; doi:10.3389/fsurg.2023.1101294)
Supplement: Supplementary file 1 [file Datasheet1.docx]

**Supplementary information**

Table 1. Treatments and relative clinical evaluations of the patient during 16 courses

| **Time** | **Courses** | **Administration** | **Treatment** | **Evaluation** |
| --- | --- | --- | --- | --- |
| 2019-7-6 | 1 | Ivgtt | Oxaliplatin | N/A |
|  |  | PO | Capecitabine |  |
|  |  |  | Tratinib |  |
|  | 2 | Same course with course 1 | | PR |
|  | 3 | Same course with course 1 | | N/A |
|  | 4 | Same course with course 1 | | SD |
|  | 5 | Same course with course 1 | | N/A |
|  | 6 | Same course with course 1 | | PD |
| 2019-12-3 | 7 | TACE | Fluorouracil | SD |
|  |  |  | Cisplatin |  |
|  |  |  | Pirarubicin+lipiodol |  |
|  | 8 | TACE | Fluorouracil | SD |
|  |  |  | Cisplatin |  |
|  |  |  | Pirarubicin+lipiodol |  |
|  |  | Ivgtt | Docetaxel |  |
|  |  | PO | Capecitabine |  |
|  |  |  | Anlotinib |  |
|  | 9 | Same course with course 8 | | PD |
| 2020-2-12 | 10 | Ivgtt | Carrelizumab | N/A |
|  |  |  | Fluorouracil |  |
|  |  |  | Oxaliplatin |  |
|  |  | PO | Anlotinib |  |
|  | 11 | Same course with course 10 | | PR |
|  | 12 | Same course with course 10 | | N/A |
|  | 13 | Same course with course 10 | | PD |
|  | 14 | Ivgtt | Carrelizumab | PD |
|  |  |  | Docetaxel |  |
|  |  |  | Cisplatin |  |
|  |  | PO | Apatinib |  |
|  | 15 | Ivgtt | Carrelizumab | PD |
|  |  |  | Docetaxel |  |
|  |  |  | Cisplatin |  |
|  |  | PO | Anlotinib |  |
|  | 16 | Ivgtt | Carrelizumab | PR |
|  |  | Gamma SBRT | 39Gy/13FX |  |

**Notice and Abbreviation index:**

PO: peros

ivgtt: intravenous drip

TACE: transcatheter arterial chemoembolization

Gamma SBRT: Gamma Stereotactic Body Radiotherapy

RECIST1.1: Response Evaluation Criteria In Solid Tumours 1.1 (2009)

CR: complete response

PR: partial response

SD: stable disease

PD: progressive disease

N/A: no available

**
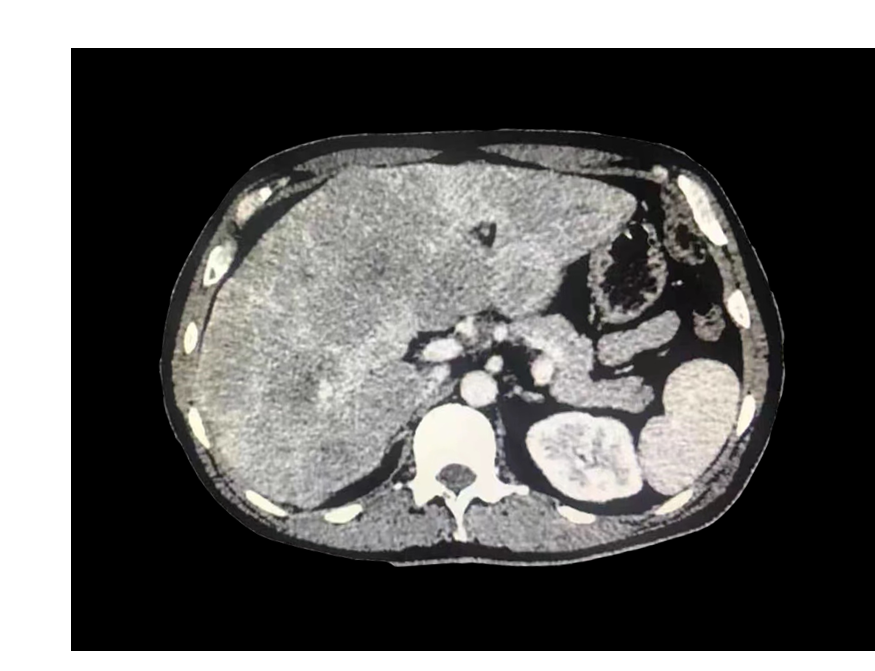
**

*Figure S1.* *CT images of liver lesions in June 2019 (before treatment), magnification image from Figure 1A.*

*
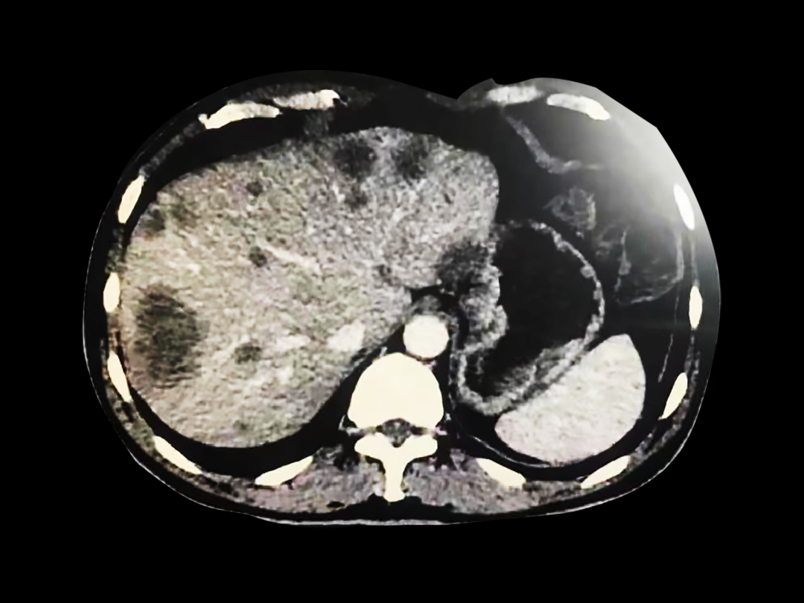
*

*Figure S2.* *CT images of liver lesions in September 28, 2019, magnification image from Figure 1B.*

*
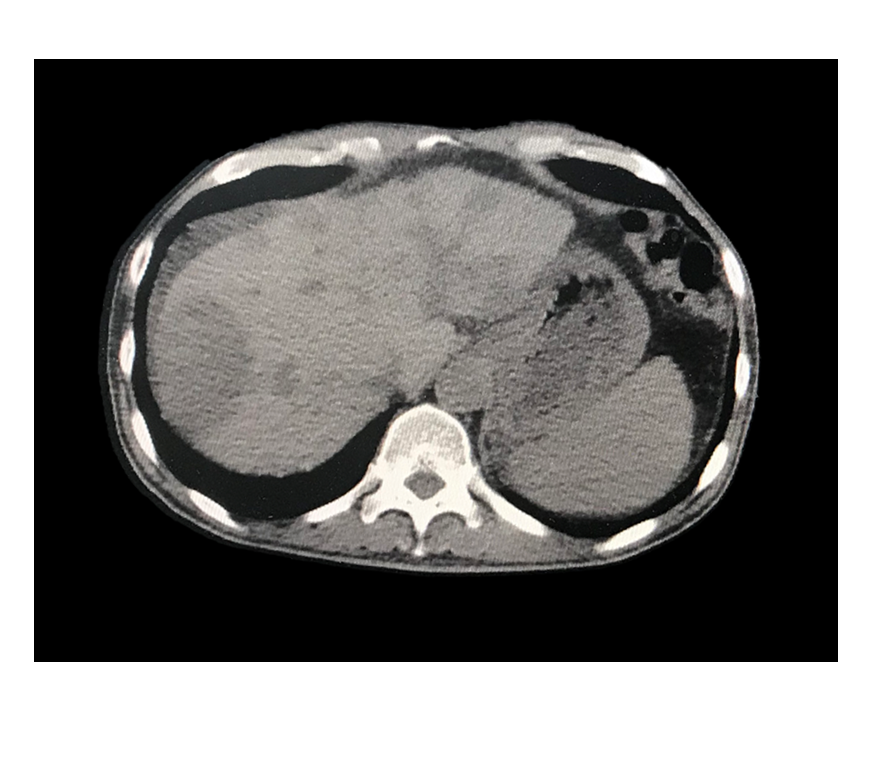
*

*Figure S3.* *CT images of liver lesions in December 23, 2019, magnification image from Figure 1C.*

*
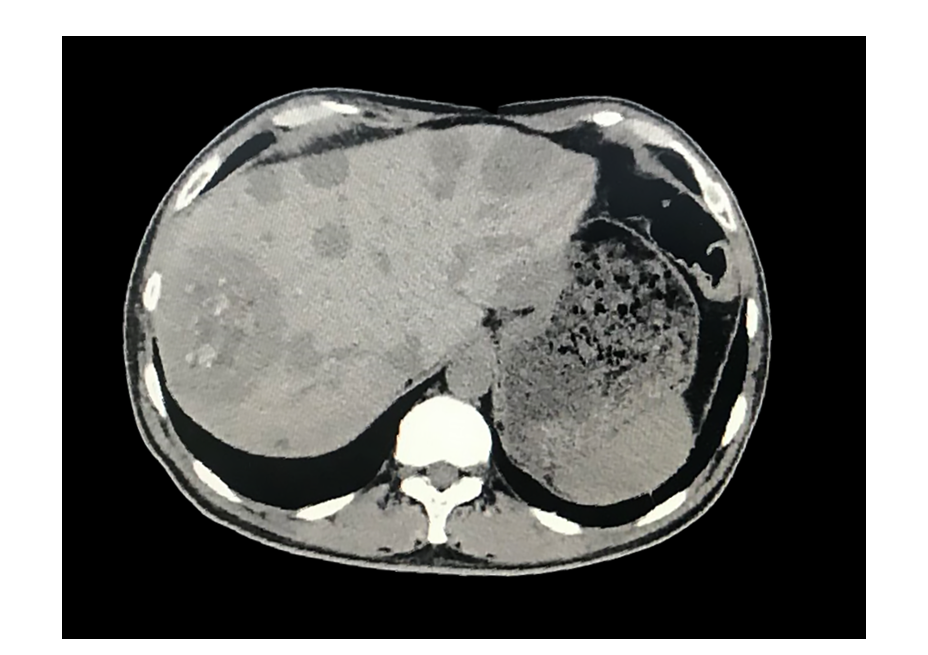
*

*Figure S4.* *CT images of liver lesions in June 9, 2020, magnification image from Figure 1D.*

*
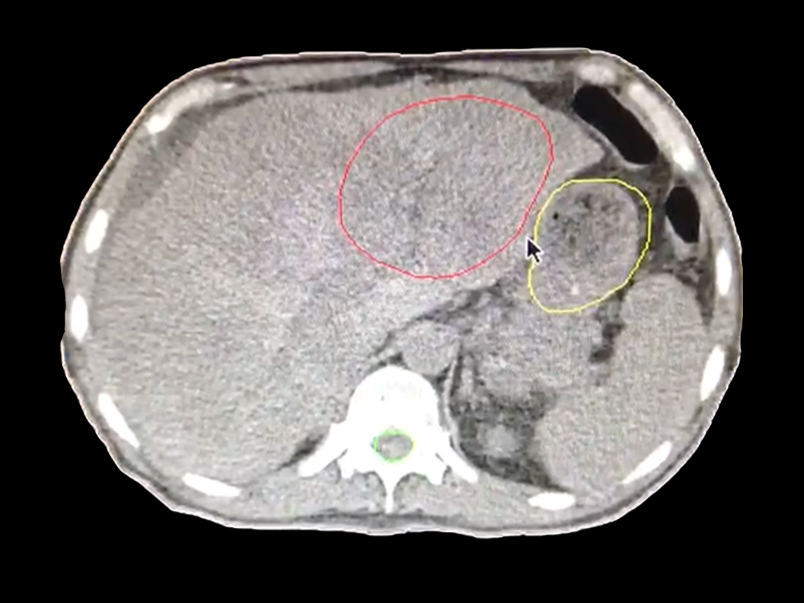
*

*Figure S5.* *CT images of liver lesions in July 20, 2020, magnification image from Figure 1E.*

*
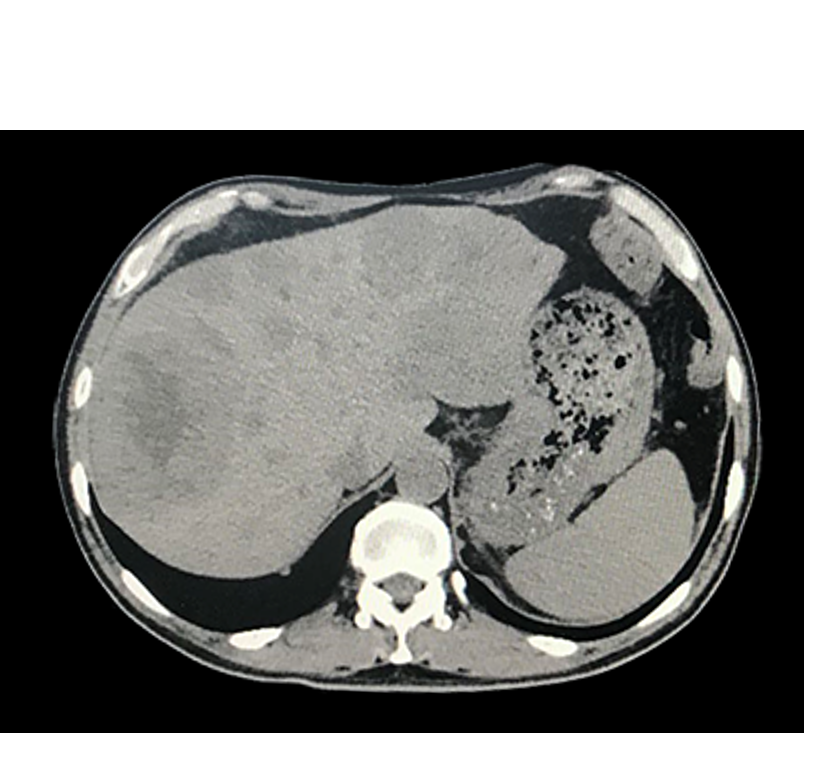
*

*Figure S6.* *CT images of liver lesions in September 4, 2020, magnification image from Figure 1F.*
